# Maternal hypertensive mother’s knowledge, attitudes and misconceptions on hypertension in pregnancy: A multi-center qualitative study in Ghana

**DOI:** 10.1371/journal.pgph.0001456

**Published:** 2023-01-06

**Authors:** Evelyn Tamma, Kwame Adu-Bonsaffoh, Adanna Nwameme, Phyllis Dako-Gyeke, Emmanuel Srofenyoh, Joyce Browne

**Affiliations:** 1 Holy Care Specialist Hospital, Accra, Ghana; 2 Department of Obstetrics and Gynaecology, University of Ghana Medical School, Accra, Ghana; 3 Julius Global Health, Julius Center for Health Sciences and Primary Care, University Medical Center Utrecht, Utrecht University, Utrecht, The Netherlands; 4 Department of Social and Behavioural Sciences, School of Public Health, University of Ghana, Accra, Ghana; 5 Department of Obstetrics and Gynaecology, Greater Accra Regional Hospital (Ridge), Accra, Ghana; Emory University, UNITED STATES

## Abstract

Hypertension in pregnancy is one of the commonest complications of pregnancy and a leading cause of maternal and perinatal morbidity and mortality globally, with the highest burden in low and middle income countries. Pregnant women’s knowledge about hypertension in pregnancy facilitates early health seeking behavior, which can result in early diagnosis and treatment. This study therefore explored the knowledge, misconceptions and attitudes of Ghanaian women who were affected by hypertension in pregnancy. A qualitative study was carried out across five referral hospitals in the Greater Accra Region of Ghana. In-depth interviews (IDIs) and focus group discussions (FGDs) were used to explore the women’s knowledge on hypertensive disorders of pregnancy (HDP), and particularly preeclampsia. Women of at least 16 years, admitted with a HDP to the maternity ward with gestational ages from 26–34 weeks were eligible for participation. The inductive approach was used to develop a code book and the dataset was coded using Nvivo version 12 software. A total of 72 women participated in the study. Fifty IDIs and 3 FGDs involving 22 women were conducted. Although most of the women had regular antenatal visits, several had never heard of “pre-eclampsia”. More common terminology used by women (i.e. “Bp”) referred to any of the hypertensive disorders (e.g. pre-eclampsia, gestational hypertension and chronic hypertension). Women also perceived that pre-eclampsia may be inherited or caused by “thinking too much”. The study revealed that the knowledge about hypertension in pregnancy is limited among the affected women despite regular antenatal attendance with some form of health education. There should be more education programs on hypertensive disorders of pregnancy including pre-eclampsia with revised strategies.

## Introduction

Maternal hypertension or hypertensive disorders of pregnancy (HDP) is a common pregnancy complication that affects approximately 10% of pregnancies [1]. HDP include gestational hypertension, pre-eclampsia (which may be classified as mild or severe) and chronic hypertension [2]. HDP are a leading cause of maternal and perinatal morbidity and mortality globally, with the highest burden reported in low- and middle-income countries (LMICs) [3, 4]. In LMICs the prevalence of pre-eclampsia is estimated from 1.8 to 16.7% [5], with highest prevalence observed among women from African descent [6].

(Severe) pre-eclampsia has early warning signs and symptoms such as headaches, blurred visions, rapidly increasing swelling of the face, hands or feet [7]. As such, a woman’s knowledge about the condition can influence early health seeking behaviour, and result in early diagnosis, treatment [8, 9] and prevent pregnancy complications including maternal and perinatal deaths. Also, other studies have shown that understanding of one’s medical condition positively influences compliance to treatment and helps prevent further complications [10, 11]. However, misconceptions and lack of knowledge can lead to misdirection in seeking other means of care other than medical treatment such as making incisions on the head of a woman as a traditional way of treating eclampsia [12]. Misconceptions previously reported include the interpretation of warning signs of pre-eclampsia as a normal occurrence during pregnancy. For example, in Ghana, some women believe that swelling of the face, hands or feet is an indication that the woman is going to have a baby boy [13]. Others include the attribution of preeclampsia as “spiritual attacks”, as described in Northern Nigeria in the belief that pre-eclampsia is caused by “isaka” (spirits) [14].

Ghana introduced focused antenatal care (FANC) in 2002 to improve the quality of antenatal care (ANC) and reduce maternal and perinatal morbidity and mortality [15]. FANC includes education about, identification and management of obstetric complications such as HDP [16], and ‘pregnancy school’ or maternal classes were introduced as part of ANC in Ghana. In these, women are grouped according to their trimester in pregnancy and educated on several health issues related to pregnancy and delivery [17, 18]. In some facilities, each pregnant woman is assigned to her own midwife who sees her every time she comes for her antenatal visit. This enables the midwife to involve partners or support persons in the care process. The pregnant women together with their partners or support persons on the other hand have the contacts of their respective midwives who they can call to report any pending health issues or seek advice [15].

Despite the high coverage of ANC from skilled birth attendants (98%) with 89% receiving the recommended 4 or more ANC visits and a 79% facility-based delivery across the nation [19], HDP remains a leading cause of maternal mortality in Ghana contributing between 18.6% - 37.0% to all maternal deaths from the periods of 2008–2018 [20–22]. Recent maternal health data reported the proportion on maternal death from HDP had double over a ten-year period [19]. There is evidence that inadequate understanding of maternal hypertension constitutes a significant barrier to accessing health facilities for treatment (delay in seeking care). Optimizing maternal health and pregnancy outcomes of maternal hypertension requires global effort to improve women’s knowledge and correct harmful misconceptions on the hypertension in pregnancy. This paper therefore explores the knowledge, misconception and attitudes of Ghanaian women who were affected by maternal hypertension, based on their experiences with care in Ghana.

## Methods

### Study design, sites and population

This is a qualitative study conducted between June 2018 to March 2019 to understand the lived experiences of who had recently been admitted to health facilities for hypertensive disorders of pregnancy. In this study, the qualitative research approach used was phenomenology. This approach describes individuals’ experiences of a specific event, situation or phenomenon [23]. Hence to gain a better understanding of the women’s knowledge, attitudes and misconceptions about hypertensive disorders of pregnancy phenomenological approach was best suited. As part of the study design in-depth interviews (IDIs) and focus group discussions (FGDs) were employed. These methods of data collection provided triangulation or multiple sources of data which enriched the overall data (data source triangulation). Whilst IDI provides an important approach of gaining relevant understanding of phenomena and exploring concepts into details, FGD generates a dynamic and interactive discourse among participants resulting a diverse participants’ experiences [24]. The study was carried out across five sites in the Greater Accra Region: Greater Accra Regional Hospital (Ridge), Korle-Bu Teaching Hospital, La-General Hospital, Lekma Hospital and Tema General Hospital. These are major referral obstetric care centers in the Greater Accra Region with referral functions, large patient load, large delivery volumes and provision of comprehensive obstetric and newborn health services. The total annual number of deliveries in all these five institutions is more than 30,000 and the estimated average incidence of HDP is about 8%. In the Greater Accra Region of Ghana where the current study was conducted, about 41% and 56% of pregnant women receive ANC from doctors and midwives/nurses respectively whilst the two groups of health workers provide about 25% and 65% assistance at delivery respectively. In Ghana, the maternal mortality ratio is estimated at 308 per 100,000 livebirths [25] and maternal hypertension account for approximately 18% [19].

The doctors and midwives are responsible for handling maternal hypertensive cases in the hospitals. An organized antenatal education is mostly done by midwives; however individualized client education and counselling are maybe conducted by the obstetric doctor. In all these hospitals early screening for preeclampsia is routinely undertaken using regular blood pressure measurement and testing for urine proteins. Low-risk women are seen monthly at the antenatal clinic. On the other hand, those who are at high risk of maternal hypertension are seen more frequently at the clinic by the doctors. Education on the condition is intensified for high-risk women so that when they are at home and experience any of the symptoms or observe any of the signs associated with the condition they can report at the hospital.

The inclusion criteria for participants were women of at least 16 years, admitted with a HDP (gestational hypertension, pre-eclampsia, superimposed pre-eclampsia on chronic hypertension and eclampsia) to the maternity ward at gestational ages from 26–34 weeks. Generally, hypertensive disorders of pregnancy disproportionally affect younger women [26]. Therefore, including women from 16 years and above will enhance the generalizability and relevance to clinical care. In addition, women who were at least 16 years of age did not require parental consent to participate in the study. The focus of this study was on women who had HDP because they had lived experiences of the condition as compared to those who had not. Also, severe early-onset pre-eclampsia before 26 weeks is associated with high maternal morbidity and very poor perinatal outcome even in high income settings [27]. However, severe preeclampsia between the gestational age of 26 to 34 weeks are key grey zones in obstetric care that needs to be elucidated. The gestational ages of all the pregnancies were confirmed through obstetric dating ultrasound scans.

### Recruitment, sampling and data collection procedures

Participants for this qualitative study were recruited through purposive sampling with the aim of achieving variety in terms of obstetric history, age and socio-economic class. Purposive sampling is a non-probability method, and appropriate for qualitative research which seeks to achieve in-depth insights regarding opinions and experiences of the population of interest, rather than achieving statistical generalizability of findings [28]. This method of sampling also allows researchers recruit only participants who can provide the needed information for a study [29]. Midwives and doctors who were part of the research team helped identify women who met the inclusion criteria. The research assistants approached and explained the study protocol to eligible candidates. Women who agreed to participate in the study were given a date and time for the interviews. The midwives and doctors in the research team were not directly involved with the clinical management of the patients. All the interviews and focus group discussions were carried out in dedicated quiet rooms at the various hospitals. Participants who had delivered and been discharged were interviewed during their six weeks postpartum follow up. Participants provided written informed consent. They were assured of confidentiality of any information they provided. Recruitment of participants continued until no new themes emerged from the data (data saturation was reached).

A female research assistant competent in qualitative interviews was recruited and trained to conduct the IDIs and FGDs (ET). She was a non-healthcare provider, with a Bachelor and Master’s degree. Her inclusion in the research team was to encourage participants to freely share their experiences with the non-medical professionals during their hospital admissions and perhaps more so than they would with a medical professional. In addition to her, a note taker (MB) with a bachelor’s degree was employed for the FGDs. The interviewer and the principal investigator (obstetrician) attended a two-day qualitative data analysis workshop at the School of Public Health, University of Ghana facilitated by social scientists from the institution.

Pilot IDIs were carried out at Korle-Bu Teaching Hospital. IDIs and the FGDs were conducted in either Twi or Ga (Ghanaian local languages), audio recorded, and lasted between 17 to 40 minutes and 80 to 120 minutes, respectively. IDIs and FGDs were guided by interview or discussion guides (S1 Appendix) which included questions like: why the respondent was admitted at the facility, whether she has ever heard of any of the HDP which is locally referred to as “Bp” and what she knows about “Bp”, among many others. Participants were provided with water and soft drinks during FGDs and given GHS 15 (about the equivalent of 2.5 USD) to cover their transportation expenses.

### Data management and analysis

All in-depth interviews and focus group discussions were transcribed verbatim from Twi and Ga into English. The interviewer checked all the transcripts for accuracy and completeness. Based on the findings and themes that emerged from the data, the code book was developed. The coding was done by the interviewer (ET) and the principal investigator (KAB) using Nvivo version 12 software.

### Ethical consideration

The study obtained approval from Ghana Health Service Ethical Review Committee and the Ethical and Protocol Review committee of the College of Health Sciences, University of Ghana (protocol number: CHS-EtM.4-P1.2/2017-2017). All collected data was handled confidentially and anonymously. Access to the data was limited to the study team. There was no disclosure of participant information to others.

## Results

Fifty IDIs and three FGDs with 22 women were conducted with a total of 72 women (Table 1). Twenty women declined to participate in IDIs because they were either too ill to participate on the day of the interview or not interested in the study and did not provide informed consent. The focus group discussions were carried out at Korle-Bu Teaching Hospital, Greater Accra Regional Hospital and Tema General Hospital. The other two hospitals did not have enough women to participate in focus group discussions. For the FGDs, 33 out of the 55 originally approached did not turn up for the discussions. The reasons some of the women gave for not turning up had to do with the trauma they experienced due to the condition or the loss of their babies.

**Table 1:** Social demographics of respondents for IDIs and FGDs.

	IDIs n = 50(%)	FGDs n = 22(%)	Total n = 72(%)
**Age (years)**			
<20	3 (6.0)	0 (0)	3 (4.2)
20–24	3 (6.0)	2 (9.1)	5 (6.9)
25–34	36 (72.0)	11 (50.0)	47 (65.3)
35+	8 (16.0)	9 (40.9)	17 (23.6)
**Marital status**			
Single	17 (34.0)	5 (22.7)	22 (30.6)
Married	33 (66.0)	13 (59.1)	46 (63.9)
Cohabitating	0 (0)	4 (18.2)	4 (5.6)
**Education**			
None	1 (2.0)	3 (13.6)	4 (5.6)
Primary	13 (26.0)	0 (0)	13 (18.1)
JHS	17 (34.0)	9 (40.9)	26 (36.1)
SHS	10 (20.0)	6 (27.3)	16 (22.2)
Vocational/Diploma	0 (0)	4 (18.2)	4 (5.6)
Tertiary	9 (18.0)	0 (0)	9 (12.5)
**Religion**			
Christian	46 (92.0)	19 (86.4)	65 (90.3)
Muslim	4 (8.0)	3 (13.6)	7 (9.7)
**Residence**			
Urban	48 (96.0)	22 (100.0)	70 (97.2)
Semi-urban	2 (4.0)	0 (0)	2 (2.8)
**Ethnicity**			
Akan	28 (56.0)	7 (31.8)	35 (48.6)
Ewe	10 (20.0)	6 (27.3)	16 (22.2)
Ga	6 (12.0)	4 (18.2)	10 (13.9)
Other	6 (12.0)	5 (22.7)	11 (15.3)
**Occupation**			
Unemployed	15 (30.0)	2 (9.1)	17 (23.6)
Trader	20 (40.0)	12 (54.6)	32 (44.4)
Designer/Seamstress	3 (6.0)	2 (9.1)	5 (6.9)
Hairdresser	2 (4.0)	3 (13.6)	5 (6.9)
Others	10 (20.0)	3 (13.6)	13 (18.1)
**Hospital**			
Korle-Bu	20 (40.0)	4 (18.2)	24 (33.3)
Ridge	16 (32.0)	10 (45.5)	26 (36.1)
La General	4 (8.0)	0 (0)	4 (5.6)
Lekma	5 (10.0)	0 (0)	5 (6.9)
Tema General	5 (10.0)	8 (36.4)	13 (18.1)
**Previous high blood pressure**			
Yes	14 (28.0)	9 (40.9)	23 (31.9)
No	36 (72.0)	13 (59.1)	49 (68.1)
**Total number of pregnancies**			
1	11 (22.0)	2 (9.1)	13 (18.1)
2–3	21 (42.0)	7 (31.8)	28 (38.9)
4–5	12 (24.0)	11 (50.0)	23 (31.9)
6+	6 (12.0)	2 (9.1)	8 (11.1)
**Total number of deliveries**			
0	6 (12.0)	0 (0)	6 (8.3)
1–2	29 (58.0)	12 (54.6)	41 (56.9)
3–5	13 (26.0)	9 (40.9)	22 (30.6)
6+	2 (4.0)	1 (4.6)	3 (4.2)

Of the women who participated, the majority were between 25–34 years (n = 47, 65.3%), married (n = 46, 63.9%) and had Junior High School (JHS) (n = 26, 36.1%) level of education. Almost all the respondents resided in the urban areas (n = 70, 97.2%) and belonged to the Christian faith (n = 65, 90.3%). Forty-nine of women had no history of previous high blood pressure (Table 1).

Important themes that emerged about the knowledge of women on pre-eclampsia or HDP and the challenges they faced during pregnancy were:

1. Knowledge on pre-eclampsia or maternal hypertension (causes, misconceptions, and antenatal education)
2. Attitude towards antenatal care visits
3. Social challenges during pregnancy
4. Recommendations to improve quality of care

### 1. Knowledge on pre-eclampsia or maternal hypertension

Almost all the respondents had heard of high blood pressure, or “BP” and knew it as a health condition commonly associated with the elderly, but not with younger people or even pregnant women. Most of them did not know that “BP” could complicate pregnancy and lead to adverse outcomes. They also described BP as when one’s heart is beating very fast, shortness of breath and blood pressure going up.

*“What I know is that when it occurs, your heart starts to beat very fast and your pressure goes up” (IDI, Single, 27 years)*

*‘I have heard about Bp before but I didn’t know that you could have BP when pregnant” (IDI, Married, 31 years)*

*”At first, I had heard of it (respondent laughing) but I didn’t know that children like us who are not really old can get it. I know the older people are those who might have this disease. (IDI, Married, 39 years)*

*“Please when they say BP*, *I don’t understand*. *I just asked someone recently and she said the heart beating very fast is BP*. *I have heard it all the time but I didn’t know that was it*” *(FGD*, *Cohabiting*, *42 years)*

Pre-eclampsia as a word was largely unknown to the participants. The terminology they were familiar with, “BP;” referred to any of the hypertensive disorders including pre-eclampsia, gestational hypertension and chronic hypertension.

*“I knew about Bp but I didn’t have it but as for pre-eclampsia, if not for the pregnancy, I have never heard of it” (FGD, Married, 42 years)*

*“I do interact with them [doctors]. I ask them questions because it was…, the condition was new to me. I was even surprised I hadn’t heard it before” (IDI, Married, 26 years)*

*“Actually, I’ve never heard of the name because it’s been a long time since I delivered. I don’t know there is a sickness like that” (IDI, Married, 28 years)*

#### Perceived causes of pre-eclampsia or maternal hypertension

Participants perceived “excessive worrying” or “thinking too much” as the main cause of pre-eclampsia, although a hereditary link was also identified. For this reason, women who were “not stressed” about anything during pregnancy were surprised when they were told they had Bp because they only associated it with those who “think a lot”.

*“They say some of the Bp is caused by too much thinking, that’s what I’ve heard. When you think too much then it comes” (IDI, Married, 41 years)*

*“I will say there are those who have it in the family so that one is fifty, fifty [fifty percent chance of inheriting hypertension or not] and there are those who when you think too much, brings it” (IDI, Married, 29 years)*

*“Honestly if I say I know something about Bp then am lying. What I know is, if you think too much, is when your Bp goes up but I don’t think about anything so I was surprised when I was told my Bp had gone up” (IDI, Married, 33 years)*

#### Experience of symptoms and signs of pre-eclampsia

Most women experienced the main symptoms of preeclampsia or maternal hypertension, but did not understand what was happening to them and did not report to health facilities. However, few women reported to hospital when they experienced severe symptoms even though their scheduled antenatal visit were not due. Participants reported having experienced headaches, swellings all over the body, dizziness and sleeplessness during their pregnancies.

*“I had headaches and I felt dizzy. I am a hairdresser and I braid hair so when I stand for long then I feel dizzy and have to sit so that makes me weak” (FGD, Married, 24 years)*

*“The pregnancy really disturbed me. I had headaches and felt weak. In the seventh month I experienced severe headaches and I couldn’t sleep at night” (FGD, Married, 28 years)*

*“My pregnancy was okay until one day I woke up with a swollen face and a swollen body. My antenatal date wasn’t due but because of the headache I was experiencing, I came to the hospital and they referred me here” (IDI, Married, 30 years)*

#### Misconceptions about the causes and treatment of pre-eclampsia or maternal hypertension

Some women perceived the condition as a spiritual one that needed to be treated by staying in prayer camps or churches, on the advice of priests or doctors.

Women indicated that the referral to prayer camps or churches had substantial financial consequences. One participant was convinced that doctors only suggested an operation as a form of treatment for financial gain.

*“The previous pregnancy*, *I felt very ill during the pregnancy to the extent that my pastor wanted me to even come around so he sees me*. *I wanted to go to the hospital but he said the sickness was a spiritual one and not a hospital issue*. *So I spent a lot of time in the church to the extent that before we had left the church*, *I had spent about 2500 Ghana cedis*. *So I was still in the church till one day when I saw I was bleeding*. *It was late and everybody was asleep in the church*. *I called my husband to come over to the church*. *When he came*, *I had delivered in the church*. *The child grew a bit but died*” *(IDI*, *Married*, *30 years)*

*“One of the doctors told me to send it to prayers [seeking divine intervention such as going to the church or prayer camp] because I have been given some drugs [BP medication] but it [BP] keeps going up and down” (IDI, Married, 41 years)*

*‘Yes, but what I don’t like is that when you come here and they are supposed to discharge you they refuse and want to force you to do operation because they will get some money from you” (IDI, Married, 41 years)*

#### Education during antenatal care

The respondents indicated they were educated on diet, grooming, exercise and danger signs in pregnancy. They were told not to eat foods with high levels of salt and spices and were encouraged to eat fruits, vegetables and foods that could boost their “blood levels” (hemoglobin levels). They were also advised to keep their bodies and environments clean and to exercise. The respondents were asked to rush to the hospital when they experienced headaches, blurred visions, swellings all over the body, bleeding, losing of liquor, abdominal pain or when they experienced any form of discomfort.

*“They taught us that when you are pregnant you have to watch your diet. For me they told me to stop eating salt because my legs were swollen so I stopped taking salt and spices” (IDI, Married, 30 years)*

*We were told to eat a lot of fruits, and also foods that increase our Hb such as plantain, kontomire [cocoyam leaves]; and also how to exercise. We shouldn’t exercise for too long. We should exercise for about 5 minutes; we should sometimes walk a bit to strengthen ourselves. (IDI, Single, 22 years)*

*“I attended my antenatal here and we were educated on the types of food to eat which will give strength to the baby. We were asked to eat fruits such as oranges and watermelon after the main course” (FGD, Married, 28 years)*

Most of the antenatal education centered on nutrition in pregnancy. Although the term “preeclampsia” or “hypertensive disorders of pregnancy” was not mentioned the mothers were educated on danger signs of the condition.

*“They taught us to keep ourselves well and not to look unkempt just because we are pregnant. Also, when you have headache report to the hospital for the doctor to see and not that we go ahead and take drugs by ourselves. Also, when your feet are swollen, do come to the hospital because sometimes we think when our feet are swollen, it means we are going to give birth to a boy. (IDI, Married, 30 years)*

A respondent who had experienced pre-eclampsia or HDP in her previous pregnancies still did not have adequate understanding of the condition and so was not compliant with suggested medical interventions. According to her, all the previous medical interventions did not produce the expected outcome since all her babies died after having a caesarean section.

*“When I conceived and went to the hospital, they said my Bp was high, for me any time am pregnant, within 7 months my Bp goes high. This morning when the doctor came to check and said it [Bp] has gone down so he will take out the child, I refused. He said that if he doesn’t remove the baby, it will cause problems for me but I refused because that is what they did and last 2 years the baby did not survive so he also said ok. This is the fourth time that they have tried to bring out the baby and as a result, I don’t get it, so for this time I will not agree” (IDI, Married, 41 years)*

### 2. Attitude towards antenatal care visits

From the narratives most of the women had regular antenatal care visits. They ensured they visited the hospital on their booked dates and whenever they experienced any form of discomfort even if it was not their appointment day.

*”When am pregnant, I attend antenatal early. Because with my husband, when I miss my period and I go and buy a pregnancy kit and test at home, he never believes the result unless he marches me to the hospital himself to verify. So as for me I start antenatal on time and I also attend on the date I’m given. When I experience any pain before my next appointment date I report at the hospital. (FGD, Married, 31 years)*

*Yes, I was more than regular because I was always in a hurry to go for antenatal care. (IDI, Married, 30 years)*

*I report on my booked dates. I came here on my booked date and I was told I had BP” (IDI, Married, 40 years)*

### 3. Social challenges during pregnancy

#### Martial or relationship problems

Some of the women reported marital or relationship problems during pregnancy such as partners change in attitude towards them and cheating. The women indicated that these problems lead to heightened stress levels, leading to an increase in their blood pressure.

*“So, I have been with the man for a long time but when the pregnancy came, his behaviour changed. He said he didn’t want to have anything to do with me but he will take care of the baby when born. Because of that I was thinking a lot. I was staying with him but ever since the pregnancy he made me pack my things and told me to come and stay with my mother” (IDI, Single, 26 years)*

*“My husband did something which I have been thinking about and I could say my husband is to be blamed for my Bp being high because he went for another woman whiles he was still with me. I told him to go and marry her in addition but he refused saying that he did not like her. But he went to marry her without informing me and when I heard it, I was very disturbed and could not sleep” (IDI, Married, 29 years)*

*“When I got pregnant, his attitude changed towards me and I was thinking he was probably cheating on me. So when am alone, I use to think a lot because when he goes to work he was coming too late and as such I was alone at home. Even when he came home and I told him we should take a walk before coming to sleep, he will tell me he is tired and that made me worry a lot” (IDI, Married, 25 years)*

#### Effect of admission on respondents and their families

Whiles on admission the women were worried about their children at home because they did not have anyone to help take care of them. Moreover, they felt the fathers couldn’t take care of the children as they would.

*“I am only worried about my two children I have left at home and I was hoping I would be discharged so I could go and take care of my children because the father can’t really take care of the children like the way a woman would. I have taken all my medication and I just hope to be discharged soon because my little child would cry when she doesn’t see me around” (IDI, Married, 28 years)*

### 4. Recommendations to improve quality of care: Creation of awareness by health care professionals and government

When asked what the healthcare providers and doctors could do to improve the quality of care for HDP, the women recommended education about the condition to prevent perinatal deaths, education on risk in future pregnancies, alleviation of fears and clearance of misconceptions. Education on diet was also recommended.

*“What the government should really do is to create awareness because I had never heard of pre-eclampsia until I had it so they should educate us on how to take care of ourselves so that the pre-eclampsia does not take place so that we can deliver exactly on time” (FGD, Married, 42 years)*

*“What I can say is that they should educate us and help us and manage it because I am scared of the prematurity. I am so scared of getting pregnant again because I have struggled to get pregnant. Because I have heard that sometimes if someone gets pre-eclampsia, when she gets pregnant again, she can have it and that it becomes her problem. I am so scared to get pregnant again. So if they can give us something at the early stages like, if you eat this food it could give you problems. So if they can help us so that the pre-eclampsia does not take place at all and even if it takes place, they can help manage the person for her to deliver at the right time. I think I will like it” (FGD, Married, 42 years)*

## Discussion

In this qualitative study, we explored the women’s knowledge and misconceptions on maternal hypertension based their lived experiences care in a tertiary care facility. Our study indicates that women’s knowledge level about HDP is low with substantial burden of misconception, even though majority of the women had regular antenatal care visits and received some form of education on diet and danger signs in pregnancy. HDPs were commonly perceived as a consequence of “thinking too much”. Also, the hypertensive mothers encountered significant social challenges during pregnancy includes partners’ change in attitude and infidelity. The women suggested that more awareness and education of the condition could be beneficial.

Antenatal care services provide the opportunity for educating pregnant women on complications of pregnancy. Overall, antenatal care attendance in Ghana is relatively high [30, 31]. Relatedly, the women who participated in this study were regular attendants who received some form of education from the maternal classes or pregnancy school sessions from their midwives. Yet, we identified significant knowledge gaps and misconceptions about the causes and management of HDP among the hypertensive mothers. For instance, the term “pre-eclampsia” was unknown to most of the women, and there is no word in the local dialects for pre-eclampsia. The acronym “BP” was the term mostly used to describe pre-eclampsia and all other types of HDP. The main cause of pre-eclampsia was identified by most women as “over thinking or worrying” and this is consistent with a recent report from Ghana by Joshi et al. [32].

Although women mentioned various alarm symptoms of pre-eclampsia that were discussed during ANC education, they had challenges in linking the symptoms (headaches, swellings all over the body, dizziness) they had experienced with HDP. This suggests the need for improvement in the antenatal education and counseling provided to women on preeclampsia and the associated causes, symptoms and treatment. Lack of adequate knowledge of HDP among pregnant women has been observed by others as well. Recent studies from Ghana showed high prevalence of inadequate knowledge of preeclampsia among pregnant women (88.4%) [9] and postpartum women diagnosed with preeclampsia or eclampsia (61.4%) [33]. It is important for midwives and doctors to engage the pregnant women more frequently to assess their understanding of the topics discussed during educational sessions or counselling. Also, counseling and health education of pregnant women should be regularly reinforced to improve their understanding on the important concepts.

In addition, suboptimal knowledge or high burden of misconceptions also affected women’s perceptions about treatment options and the need for medical intervention. For instance, one woman refused an emergency cesarean section due to the fear of perinatal death. Other women perceived HDP to be a spiritual condition which required spiritual treatment, causing a delay in seeking medical care. The role of traditional, religious and spiritual conceptions in HDPs have also been described by other researchers. In Northern Nigeria, the belief that pre-eclampsia is caused by “isaka” (spirits) has been reported by Osungbade and Ige [14]. A study from Ghana revealed that pregnant women together with their care providers commonly expressed the need for spiritual protection, which in some instances involved the women being kept in churches or prayer camps throughout their entire period of pregnancy until delivery [34].

The high occurrence of knowledge gaps and misconceptions suggests that the delivery of antenatal education could be more effective, and should include reference to the terms used by women (“BP”) and clinicians (hypertension, pre-eclampsia and eclampsia), and cover common misconceptions. This can also facilitate earlier recognition of symptoms and report at health facilities on time for early interventions. Education could be extended to other places where women and their families receive information, such as religious institutions, secondary schools and tertiary institutions. Allowing partners and support persons to receive information about healthy pregnancy and possible complications and their consequences, could also provide women with the necessary support through their treatment and minimize the challenges they experience. In addition, women who develop HDP and their families should also receive sufficient education to understand the disease and possible detrimental maternal and perinatal effects. Importantly, women themselves recommended that sensitization/education programs on pre-eclampsia should be adequately embarked on.

Most women with HDP indicated their experiences of social challenges. These include partner’s change in attitude, infidelity and misunderstanding or arguments, similar to observations from a study conducted in south-western Nigeria [12]. These challenges made the women more worried, anxious or in-secure. In addition, women indicated great concerns about their young children they left behind at home during admission.

One of the main strengths of this study is that it was multi- centered across five study sites in the Greater Accra region of Ghana. In addition, the qualitative design provided insight into the perceptions of women purposively sampled from different backgrounds hence enriching the findings of the study. The main limitation of the study is that it was carried out in only one region in Ghana and the findings may not be generalizable to other settings. In addition, the use of only one interviewer could have introduced some form of bias in the interviewing technique. Also, we excluded non-hypertensive mothers from the study and this might have skewed our findings to a one-sided perspectives from the hypertensive mothers. Inclusion of the normotensive mothers would have resulted in a more comprehensive overview of women’s knowledge and misconceptions about maternal hypertension. However, our main objective was to explore women’s understanding of maternal hypertension based on their experiences with the condition and how they were treated. Another important limitation relates to the high number of eligible women who could not participate in the interviews for various reasons (20 out of 70 and 33 out 55 women for the IDI and FGD respectively). The non-participation of these potential participants may have impacted negatively on the reported study findings. However, data saturation was reached when there was no emergence of new themes from the interviews indicating that all the relevant themes were covered.

## Conclusion

Our study has revealed limited knowledge and high misconceptions on hypertension in pregnancy among pregnant women admitted with HDP, even though the majority were regular antenatal care attendants who received some form of education during pregnancy. In addition, several social and financial challenges encountered by women with HDP were identified. Given the role of delayed recognition of symptoms and initiation of treatment in the occurrence of adverse maternal and perinatal outcomes, improved education programs during pregnancy and after HDP diagnosis is recommended. All pregnant women should be educated comprehensively on the risk factors for maternal hypertension and the associated complications. The term “preeclampsia” needs to be actively mentioned during antenatal educational sessions. In addition, women and their families should be provided with enough information on the symptoms and signs to the preeclampsia. Lastly, further education and counselling should be provided on clinical features of worsening or severe disease, treatment principles and adherence to treatment protocols including the need for hospital admission.

## Supporting information

S1 Appendix Guide for in-depth interview and focus group discussion. (DOCX)
