# Supplementary material for: Maternal hypertensive mother’s knowledge, attitudes and misconceptions on hypertension in pregnancy: A multi-center qualitative study in Ghana
Source: PLOS Glob Public Health. 2023 Jan 6;3(1):e0001456. doi: 10.1371/journal.pgph.0001456 (PMC10021865; doi:10.1371/journal.pgph.0001456)
Supplement: S1 Appendix — (DOCX) [file pgph.0001456.s001.docx]

**Supporting information**

**S1 Appendix: Guide for in-depth interview and focus group discussion**

1. Description of pregnancy experience including complications or problems.
2. Can you tell me something about your pregnancy?
3. Did you have any problems/complications during this pregnancy?
4. Socio-economic challenges during pregnancy
5. Did you have any issue that bothered you and made you think a lot during pregnancy, like financial problems, marital problems, job issues or living conditions, etc?
6. Antenatal care experience
7. Did you seek antenatal care during pregnancy? If yes, where and how often? If no, why not?
8. Can you describe what happens when you visit the antenatal clinic?
9. Did the doctor or midwife discuss the results of your lab tests or scans with you?
10. Do you know of pregnancy school/ maternal class?
11. Did you attend any of the maternal classes/ Did you receive any form of education during your antenatal care visits?
12. Knowledge on Bp/ hypertension
13. Have you ever heard of Bp/ hypertension? If yes where?
14. What do you know about Bp/ hypertension?
15. Facility based-delivery experience
16. Why were you admitted at this facility?
17. Can you describe what happened after you got here?
18. How did your admission here affect you and your family?
19. Recommendations to improve the quality of care of women presenting with hypertensive disorders of pregnancy (HDP)?
20. What should the doctors do to help improve the quality of care of women presenting with HDP?
21. What should the midwives/nurses do to help improve the quality of care of women presenting with HDP?
22. What should the government do to help improve the quality of care of women presenting with HDP?
